# Influence of Mucoadhesive Polymers on Physicochemical Features and Biocompatibility of Collagen Wafers

**DOI:** 10.1021/acspolymersau.5c00010

**Published:** 2025-05-01

**Authors:** Ioana Luca, Mădălina Georgiana Albu Kaya, Irina Titorencu, Cristina-Elena Dinu-Pîrvu, Maria Minodora Marin, Lăcrămioara Popa, Ana-Maria Rosca, Aurora Antoniac, Valentina Anuta, Răzvan Mihai Prisada, Durmus Alpaslan Kaya, Mihaela Violeta Ghica

**Affiliations:** † Faculty of Pharmacy, Department of Physical and Colloidal Chemistry, 367123 “Carol Davila” University of Medicine and Pharmacy, 6 Traian Vuia Street, 020956 Bucharest, Romania; ‡ Innovative Therapeutic Structures Research and Development Center (InnoTher), “Carol Davila” University of Medicine and Pharmacy, 6 Traian Vuia Street, 020956 Bucharest, Romania; § Division of Leather and Footwear Research Institute, Department of Collagen, National Research and Development Institute for Textiles and Leather, 93 Ion Minulescu Street, 031215 Bucharest, Romania; ∥ Cell and Tissue Engineering Department, “Nicolae Simionescu” Institute of Cellular Biology and Pathology, 8, B.P. Hasdeu Street, 050568 Bucharest, Romania; ⊥ Faculty of Chemical Engineering and Biotechnology, Advanced Polymer Materials Group, 343067 National University of Science and Technology POLITEHNICA Bucharest, 1–7 Polizu Street, 011061 Bucharest, Romania; # Faculty of Material Science and Engineering, Department of Metallic Materials Science and Physical Metallurgy, National University of Science and Technology POLITEHNICA Bucharest, 313 Splaiul Independentei Street, District 6, 060042 Bucharest, Romania; ∇ Faculty of Agriculture, Department of Field Crops, 52987 Hatay Mustafa Kemal University, 31034 Antakya-Hatay, Turkey

**Keywords:** collagen, hydroxypropyl methylcellulose, carbomer, wafer, biocompatibility

## Abstract

The aim of this study was to develop and characterize some freeze-dried wafers based on collagen and two mucoadhesive polymers, namely, hydroxypropyl methylcellulose (HPMC) and Carbomer 940 (CBM). The wafers were obtained by lyophilization of the corresponding hydrogels, which were evaluated by circular dichroism in order to investigate mucoadhesive polymers’ influence on collagen’s secondary structure. The obtained freeze-dried wafers were characterized by FT-IR spectroscopy, thermogravimetric analysis (TGA), scanning electron microscopy (SEM), contact angle measurements, and water uptake capacity. Furthermore, biocompatibility assessment was performed by evaluating the impact of freeze-dried wafer extracts on cell viability, morphology, and migration capacity. Circular dichroism showed more significant changes in the secondary structure of collagen associated with the addition of Carbomer 940. The FT-IR spectra displayed specific peaks for collagen and the two mucoadhesive polymers. SEM images illustrated a microporous structure for both collagen and Carbomer 940, while HPMC displayed a more sheet-like structure. The addition of HPMC increased the thermal stability of collagen, while Carbomer 940 had a negative impact on the samples’ thermal stability. Contact angle measurements and water uptake capacity showed good hydrophilicity of the wafers. Except for CBM 100%, all samples supported the viability of human fibroblasts and did not have any inhibitory effect on cell migration capacity, demonstrating good biocompatibility, which is an essential attribute in developing drug delivery supports intended for mucosal applications.

## Graphical abstract

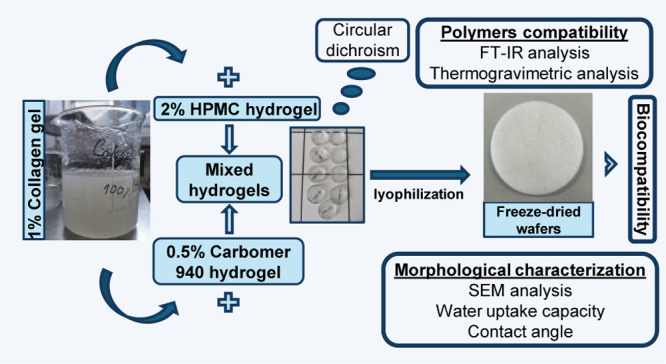

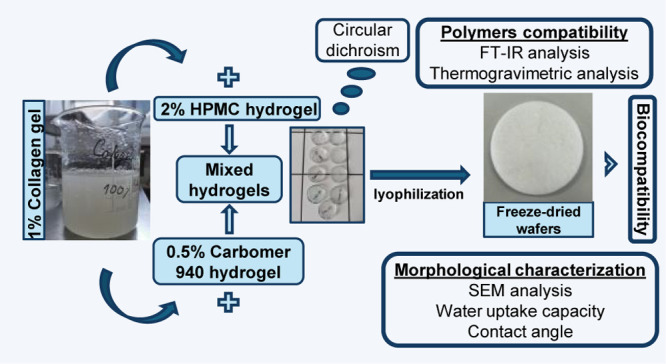

## 1 Introduction

Interest in mucosal drug delivery has been growing in recent years. Examples of mucous membranes targeted for localized drug delivery can be found in areas like the oral cavity, respiratory tract, nose, eyes, rectum, and vagina. Drug administration through mucosal membranes has several advantages compared to oral or parenteral administration routes: mucous membranes have a rich blood supply, facilitating drug absorption directly into the bloodstream and allowing for faster onset of action. Drugs administered through various mucous membranes avoid the digestive system and first-pass metabolism in the liver, which can be useful in the case of drugs that are poorly absorbed or deactivated in the gastrointestinal tract. Thus, through the localized treatment approach, increased bioavailability can be achieved, maximizing the therapeutic effect using smaller doses.^1–3^

Despite their many advantages, a well-known drawback of transmucosal drug delivery systems is their short residence time at the mucosal site due to various defense and self-cleaning mechanisms found at the administration site. In this regard, an important prerequisite in the development of these systems is that they possess mucoadhesive properties.^4^ Mucoadhesion plays a crucial role in enhancing the residence time of drug delivery systems at the site of action, improving drug absorption, and ensuring targeted drug release over an extended period of time.^5,6^ One essential aspect regarding mucoadhesive drug delivery supports is represented by the interaction between the mucoadhesive material and the mucus layer.^7^ Mucus is a complex gel-like substance composed of complex glycoproteins, also known as mucins, water, which is the predominant component, and various other constituents, such as inorganic salts or lipids. Among its many functions, it has a protective role for the mucosal surface and ensures lubrication.^8^ The most important components of mucus that are responsible for its adhesive, cohesive, and gel-forming properties are mucin glycoproteins, which have different structures and are encoded by different MUC genes depending on the body area.^8–10^

Polymers play a key role in the development of mucoadhesive formulations because they are able to attach to the mucosal tissue and mucus layer through physical or chemical bonding. There are two main categories of mucoadhesive polymers, namely those of natural origin (e.g., chitosan, alginate, gelatin, gums) and synthetic polymers (e.g., polyacrylates, cellulose derivatives, poly­(vinylpyrrolidone), poly­(vinyl alcohol)).^11,12^ Hydroxypropyl methylcellulose (HPMC) and poly­(acrylic acid) are two widely used mucoadhesive polymers in transdermal and mucosal drug delivery systems due to their favorable properties that can maximize the performance of various pharmaceutical formulations.^13–15^ HPMC is a versatile, biocompatible, water-soluble cellulose derivative often used in controlled release formulations, with many applications in oral, topical, and mucosal drug delivery.^16,17^ In solution, it remains stable across a wide range of pH levels, and its nonionic character makes it unlikely to interact with incorporated drugs.^18^ Poly­(acrylic acid) polymers, also known as carbomers or carbopols, are known for their pronounced mucoadhesive character, being capable of forming highly viscous gels upon neutralization due to their pH sensitivity.^19,20^ Moreover, carbomers are stable upon temperature variations and are great candidates for achieving controlled release.^21,22^ Based on their advantageous features, they have been found suitable for localized drug delivery, especially for topical and mucosal applications.^23^ In this regard, taking into account their well-established mucoadhesive character, HPMC and Carbomer 940 (CBM) were chosen for the present study.

Another important attribute for supports intended for mucosal drug delivery is their biocompatibility, taking into account that such systems must interact safely with the mucosal surface without triggering adverse immune responses, irritation, or toxicity.^2,24,25^ An important category of materials considered in the development of biocompatible drug delivery systems is natural polymers, which are increasingly being explored for their use in drug delivery supports due to their biocompatibility, biodegradability, and low immunogenicity.^26,27^ Collagen is a natural polymer of animal origin, with many favorable features such as biocompatibility, versatility, safety, and regulation of wound healing and re-epithelialization processes. It is the main component of the extracellular matrix and has an important role in maintaining the structural integrity of different tissues and organs.^28–32^ Being a natural component of connective tissues, collagen is highly biocompatible without causing local irritation or rejection. It also supports cell adhesion and migration, which is very important for tissue repair and regeneration.^33,34^ Moreover, due to its high versatility, it can be easily processed into various forms such as films, hydrogels, sponges, or scaffolds^34,35^ and has been used either alone or in combination with other polymers for various applications such as drug delivery,^32^ regenerative medicine (corneal, urethral, bone regeneration),^36,37^ wound healing,^30,38^ burns management,^39,40^ dentistry,^41^ gynecology, periodontal therapy or cancer treatment,^32^ among others. While collagen is a great candidate for a wide range of biomedical applications, there are some challenges associated with its use in drug delivery, such as its poor mechanical strength and thermal stability, which can lead to the alteration of drug delivery supports. To address these challenges, collagen can be combined with different natural and synthetic polymers in order to improve its properties.^32^

Sponges, also known as freeze-dried hydrogels or wafers, are solid, porous structures that have the ability to absorb large amounts of fluids or drugs within their matrix.^42^ The drug release from spongious matrices is controlled by a combination of mechanisms, which include diffusion, swelling, and erosion.^43^ Wafers can be designed to deliver drugs directly to a specific area, reducing systemic side effects by limiting drug exposure to a targeted site, and are already being used in the treatment of brain cancer.^44^ Spongious matrices are being studied and developed for several applications, such as drug delivery, wound healing, and regenerative medicine, and represent promising drug delivery supports.^45–48^

To the best of our knowledge, no other studies have aimed to develop and evaluate collagen-based wafers with either HPMC or Carbomer 940 as supports for mucosal drug delivery. Two studies used the combination of collagen and HPMC, but with the aim of obtaining either films^49^ or transparent membranes for corneal regeneration.^37^ Thus, the aim of this study was to evaluate the compatibility between a natural polymer (collagen) and two mucoadhesive polymers (HPMC and Carbomer 940) used in different ratios in the development of some hydrogels and corresponding freeze-dried wafers, as potential drug delivery supports for mucosal administration. The wafers were obtained by lyophilization of the corresponding hydrogels, which were evaluated for polymer compatibility using circular dichroism. In order to evaluate wafer morphology, scanning electron microscopy (SEM), contact angle measurements, and water uptake capacity evaluation were performed. To further assess the polymer compatibility in the obtained wafers, Fourier-transform infrared (FT-IR) spectroscopy and thermogravimetric analysis (TGA) were employed. The compatibility between collagen and mucoadhesive polymers is very important in order to further develop an ideal support for mucosal drug delivery with controlled degradability that combines the favorable features of selected polymers with the advantages of spongious matrices as dosage forms.

## 2 Materials and Methods

### 2.1 Materials

Type I collagen gel with a concentration of 2.38% (w/w) in acidic pH was extracted from calf hide using the technique currently employed at the Collagen Department of the Division of Leather and Footwear Research Institute from Bucharest, Romania. Briefly, the calf derma was treated with alkali solution (pH 12); after swelling, the fat and noncollagenous protein were mechanically removed and then washed until neutralization (pH 7); the obtained derma was then solubilized in acidic solution and precipitated with NaCl solution. After resolubilization and purification, the collagen gel was obtained. Carbomer 940 was obtained from Fagron (Greece), and hydroxypropyl methylcellulose (HPMC) was purchased from Fluka (Steinheim, Germany). Sodium hydroxide (NaOH) and triethanolamine (TEA) were obtained from Merck (Germany). The water used was ultrapure, type I water with a resistivity at 25 °C of 18.2 MΩ·cm and total organic carbon (TOC) less than 5 ppb, and was obtained with a Milli-Q EQ 7008 water purification system from Merck Millipore.

#### 2.1.1 Preparation of Individual Hydrogels

At first, three individual hydrogels were prepared. A 1 M sodium hydroxide (NaOH) solution was added under mechanical stirring to the initial 2.38% collagen gel (acidic pH), with the aim of obtaining a 1% (w/v) collagen gel (COL) with a pH value between 7.2 and 7.4, which was further used in the preparation of combined matrices. For the preparation of 0.5% (w/v) carbomer gel (CMB), 0.5 g of Carbomer 940 was gradually added to the required amount of distilled water under magnetic stirring. The 0.5% carbomer solution was then neutralized using 0.2 mL of triethanolamine, and a transparent carbomer hydrogel was obtained after mechanical stirring. In order to obtain a 2% (w/v) hydroxypropyl methylcellulose (HPMC) hydrogel, the calculated amount of HPMC was gradually brought into a beaker containing 80 °C distilled water and maintained in a water bath under mechanical stirring until complete homogenization. The remaining amount of distilled water was brought to 4 °C and added to the hot polymeric solution, which was further maintained in an ice bath under mechanical stirring to ensure proper polymer hydration. The hydrogels were kept in a refrigerator for further use.

#### 2.1.2 Preparation of Freeze-Dried Wafers

The individual hydrogels were cooled to room temperature. The combined hydrogels were obtained by mixing 1% collagen gel (pH = 7.2–7.4) with either 0.5% Carbomer 940 or 2% HPMC hydrogels in various proportions, which are presented in Table 1, using a mechanical stirrer DHL (Velp Scientifica), stirring for 5 min at 1000 rpm for each sample.

**Table 1:** Percentage Composition of the Combined Hydrogels

code	collagen, %	HPMC, %	carbomer 940, %
COL 100%	100	0	0
COL-HPMC1	75	25	0
COL-HPMC2	50	50	0
COL-HPMC3	25	75	0
HPMC 100%	0	100	0
COL-CBM1	75	0	25
COL-CBM2	50	0	50
COL-CBM3	25	0	75
CBM 100%	0	0	100

The hydrogels from Table 1 were lyophilized using a Delta LSC 2-24 Martin Christ lyophilizer (Osterode am Harz, Germany) according to the 48 h program displayed in Figure 1.

**Figure 1:**
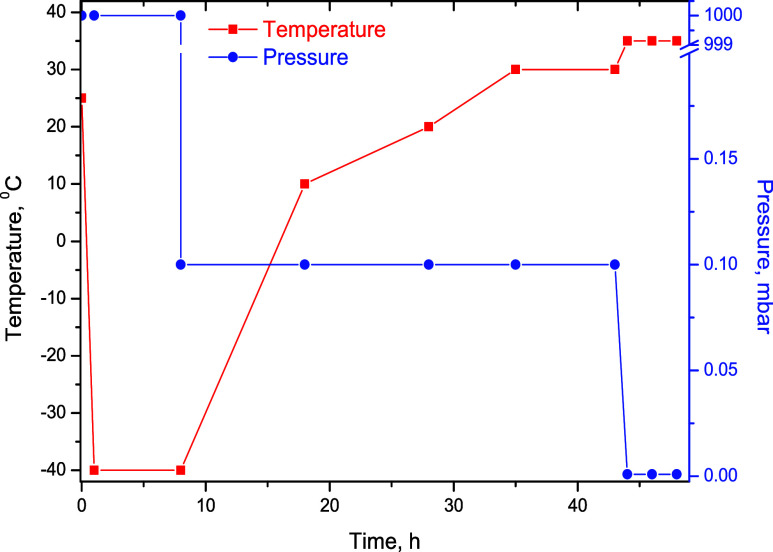
Freeze-drying diagram.

All hydrogels were dried to obtain sponges (wafers) by the lyophilization (freeze-drying) method. The hydrogels were poured into 5.2 cm diameter glass Petri dishes and then placed in a precooled lyophilizer at −40 °C. The lyophilization consists of three steps: freezing, main freeze-drying, and final freeze-drying. The freezing lasted 8 h at −40 °C, followed by main freeze-drying in 3 steps, with a constant pressure of 0.1 mbar and increasing from −40 to +10 °C in 10 h, to +20 °C in another 10 h, to +30 °C in 7 h, and keeping at that pressure and temperature for another 7 h. The final freeze-drying process lasted for 6 h at 35 °C and 0.001 mbar.

### 2.2 Methods

#### 2.2.1 Circular Dichroism (CD)

To investigate the preservation of the collagen triple-helical structure, either alone or in the presence of HPMC and Carbomer 940 after combined hydrogel preparation, circular dichroism was used. The CD spectra were obtained with a Jasco Model J-1500 spectrophotometer. Briefly, 2 mL of a polymeric aqueous solution was inserted into a cylindrical quartz cuvette with a path length of 10 mm. Spectra acquisition was done by wavelength scans performed in the 250–190 nm spectral range, with a scan rate of 100 nm/min.

#### 2.2.2 Fourier-Transform Infrared (FT-IR) Spectroscopy

The spectral characteristics of the freeze-dried wafers were obtained by FT-IR spectroscopy using a JASCO FT-IR 4200 spectrometer. Data acquisition was carried out in the 4000–800 cm^–1^ spectral range, with a resolution of 4 cm^–1^. Each obtained spectrum is an average value of 30 scans per sample.

#### 2.2.3 Scanning Electron Microscopy (SEM)

Scanning electron microscopy (SEM) was performed using a Quattro S scanning electron microscope (Thermo Fisher Scientific, OR). The microstructure of the samples was investigated in a low-vacuum operating mode without being coated with a conductive layer.

#### 2.2.4 Thermogravimetric Analysis (TGA)

The thermal stability of the obtained freeze-dried wafers was investigated by using a NETZSCH TG 209F1 Libra thermobalance (Selb, Germany). Samples with a mass of 3.83–4.53 mg corresponding to each wafer were placed in aluminum pans. The analysis was conducted under a nitrogen atmosphere (flow rate of 20 mL/min). The mass change of the samples was recorded in the 25 to 700 °C temperature range, and a heating ramp of 10 °C/min was employed.

#### 2.2.5 Contact Angle Measurements

Contact angle measurements were performed at room temperature using a KSV Cam 101 Scientific Instrument equipped with a digital camera (Helsinki, Finland). The contact angle method was employed using distilled water as the liquid. The drop shape was monitored with a digital camera, and the water contact angle values were recorded. The experiments were performed in triplicate.

#### 2.2.6 Water Uptake Capacity of Freeze-Dried Wafers

The water uptake capacity of the formulated wafers was investigated. The analysis was carried out using distilled water. Initially, the wafers were weighed in their dry form. Then, pieces of 1 cm^2^ were brought to a 24-well microplate, and 2 mL of distilled water was added on top of the wafers in each compartment. Over a period of 72 h, the wafer samples were withdrawn from the water at specific time points, kept hanging for 30 s, and weighed. To evaluate the water uptake capacity, the following formula (eq 1) was used:

1 $$w\mathrm{ater up}\mathrm{take capacity}\;\left(g/g\right)=\left(W_{t}-W_{i}\right)/W_{i}$$

where *W*_t_ represents the weight of the swollen wafer at specific time points, and *W*_i_ is the initial weight of the wafer in the dry state. The experiment was conducted in duplicate.

#### 2.2.7 Assessment of Biocompatibility of the Freeze-Dried Wafers

The freeze-dried wafers were incubated in a complete culture medium, which was low-glucose (1 g/L) Dulbecco’s modified Eagle’s medium (DMEM) (Sigma-Aldrich, St. Louis, MO) supplemented with 10% (v/v) fetal bovine serum (FBS) (Gibco BRL, Gaithersburg, MD), and 100 IU/mL penicillin, 100 μg/mL streptomycin, and 50 μg/mL neomycin (Sigma-Aldrich, St. Louis, MO) at a 2 mg/mL ratio for 7 h/37 °C, under stirring. Next, the samples were centrifuged (300 g/5 min), and the supernatant was filtered (0.2 μm pore size). Then, the effect of this extract on the viability of human adult fibroblasts was assessed, as well as its impact on cell morphology and function (migration capacity). The chemotactic properties of the tested freeze-dried wafers were assessed by an xCELLigence RTCA system (Acea Biosciences, Inc.). Human adult fibroblasts were obtained as previously described^50^ and cultured in complete culture medium, as described above, at 37 °C and 5% CO_2_.

Cells were seeded in complete medium either on 96-well plates (for viability assessment) or on glass cover slides (for morphology evaluation) at a density of 1 × 10^4^ cells/cm^2^ and left to adhere. Then, the cells were washed with phosphate-buffered saline (PBS), and the extracts obtained from the freeze-dried wafers as well as complete culture medium as a control were added. Viability was evaluated in triplicate using the XTT assay (reagents from Thermo Fisher Scientific, Waltham, MA) 24, 48, and 72 h after the addition of the extracts. The cells grown on cover slides were fixed with 4% paraformaldehyde and 0.1% Triton X-100 (Sigma-Aldrich, St. Louis, MO) at the same time points. The actin filaments were stained with phalloidin-FITC according to the manufacturer’s instructions (Thermo Fisher Scientific, Waltham, MA), and the nucleus was stained with DAPI (Sigma-Aldrich, St. Louis, MO) and detected under a Zeiss Observer D1 microscope.

The effect of freeze-dried wafers on cell migration capacity was evaluated by a scratch test. Fibroblasts were seeded in complete medium on 96-well plates (1 × 10^4^ cells/cm^2^). After reaching confluence, 24 h before the test, cells were serum-starved. A lesion was performed on the monolayer using a 200 μL pipette tip, and the cells were incubated as described above, using 5 biological replicates. The cells were photographed proximately after adding the extracts (0 h) and at 16 h. Cell migration was evaluated by quantifying the scratched area covered by the cells using ImageJ software (NIH, Bethesda, MD) and expressed as a percentage of coverage of the scratched area.

The chemotactic properties of the freeze-dried wafer extracts were evaluated using the xCELLigence RTCA system. Briefly, cell suspensions (10^5^ cells/100 μL medium) of fibroblasts were added onto the upper well of the CIM plate (a modified Boyden chamber, made by a polyethylene terephthalate membrane with 8-μm pores and microelectrodes onto their inner face that generate an impedance signal upon contact with cells). Extracts obtained from COL and COL-HPMC3 were assessed against complete culture medium as a positive control and medium without serum as a negative control in triplicate. Cell migration through the membrane was monitored in real-time for up to 24 h.

## 3 Results and Discussion

### 3.1 Circular Dichroism (CD)

Circular dichroism was used in order to investigate the possible occurrence of conformational changes in collagen’s structure, caused by the interaction with the mucoadhesive polymers; this technique offers valuable information about proteins’ structural integrity.^51,52^ Each type of structure corresponds to a different CD spectrum; for example, proteins with an α-helical structure display two negative bands at 222 and 208 nm, respectively, together with a positive band around 191–193 nm. Considering its triple-helical structure, collagen has a distinctive CD spectrum, resembling that of poly-l-proline II, showing a broad negative band with a minimum in the 190–200 nm region, a less intense positive band around 220–230 nm, and an intersection point corresponding to zero ellipticity, situated around 212–215 nm.^53–55^ The CD spectra obtained for the prepared collagen-based hydrogels are presented in Figure 2.

**Figure 2:**
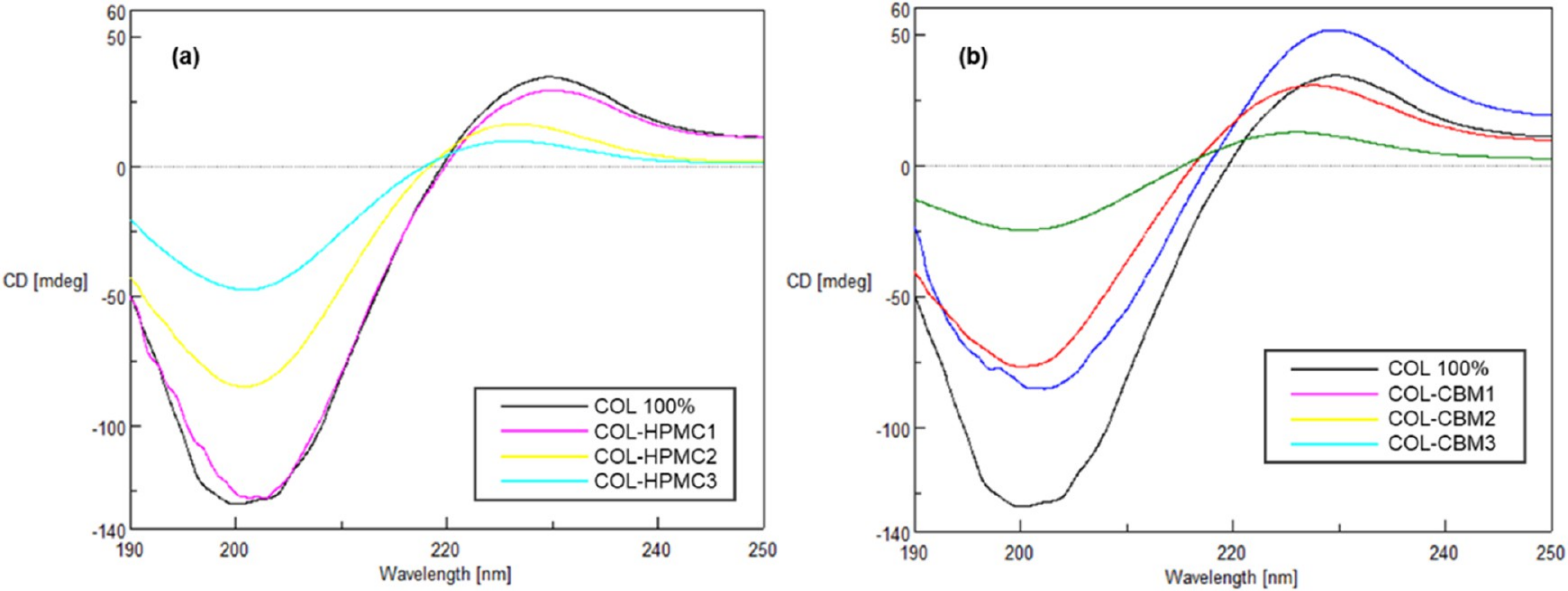
CD spectra of (a) COL-HPMC hydrogels and (b) COL-CBM hydrogels.

The CD spectra obtained for the COL 100% sample were in accordance with literature data, displaying a positive band with a maximum situated at 229.7 nm and a broad negative band with a minimum at 200.025 nm. For the three samples consisting of collagen and HPMC, combined in different ratios (COL-HPMC1, COL-HPMC2, and COL-HPMC3), a decrease in ellipticity can be observed, directly correlated with the increasing proportion of HPMC in the sample. The CD spectra of the COL-HPMC1 sample (75% collagen and 25% HPMC) are very similar to those of COL 100%, with a positive absorption band situated at 229.775 nm and a negative absorption band at 201.575 nm. COL-HPMC2 and COL-HPMC3 samples showed absorption bands with similar maximum and minimum values, compared to the CD spectra of COL 100%, but showed a gradual decrease in ellipticity for both positive and negative absorption bands. Regarding the samples consisting of collagen and Carbomer 940 (COL-CBM1, COL-CBM2, COL-CBM3), combined in different ratios, a similar trend can be noted, with a decrease of both positive and negative absorption bands, together with increasing Carbomer ratio in the sample. The only exception to this particular trend was the COL-CBM1 sample, which showed a decrease in the negative absorption band but an increase of the positive band, compared to COL 100%. All three samples showed a positive absorption band with a maximum situated between 226.3 and 229.5 nm and a negative absorption band with a minimum between 200.075 and 202 nm. The most obvious decrease in ellipticity can be observed for the sample containing 75% Carbomer 940 and 25% collagen (COL-CBM3). Among the COL-CBM samples, none of them showed high similarity to COL 100% in terms of ellipticity, as noted for the COL-HPMC1 sample. The highest denaturation was observed for COL-HPMC3 and COL-CBM3 samples, but despite the decrease recorded for positive and negative absorption bands, they can still be noticed in the CD spectra, showing that complete denaturation of collagen did not occur.

### 3.2 Fourier-Transform Infrared (FT-IR) Spectroscopy

FT-IR analysis was used in order to assess the potential changes that occurred in the secondary structure of collagen and to study interactions that arise in the obtained polymeric blends. This analysis provided information about the compatibility between collagen and two mucoadhesive polymers, based on the occurrence and intensity of the characteristic absorption bands.^56,57^ The FT-IR spectra of the COL-HPMC and COL-CBM samples are displayed in Figures 3 and 4, respectively.

**Figure 3:**
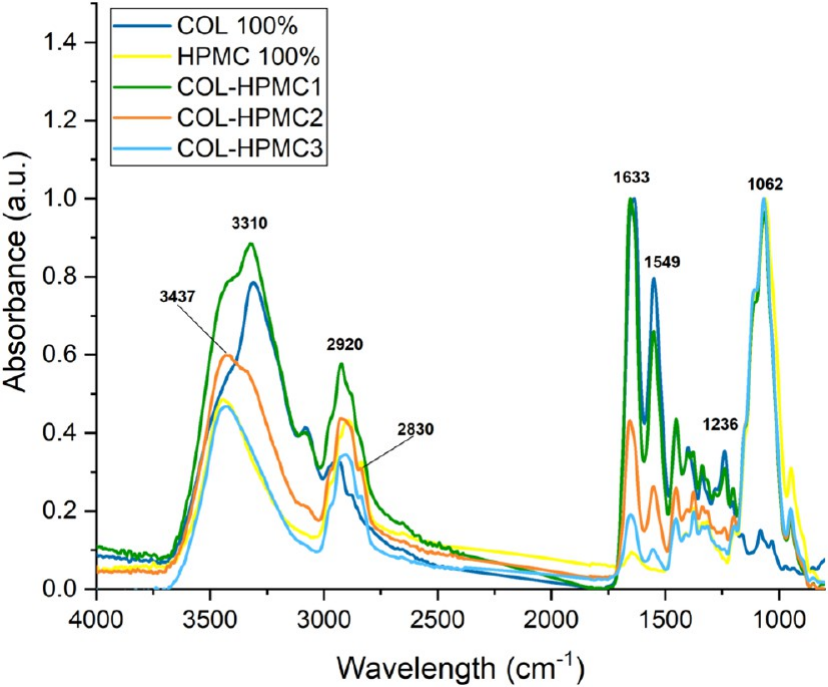
FT-IR spectra of COL 100%, HPMC 100%, and COL-HPMC samples.

**Figure 4:**
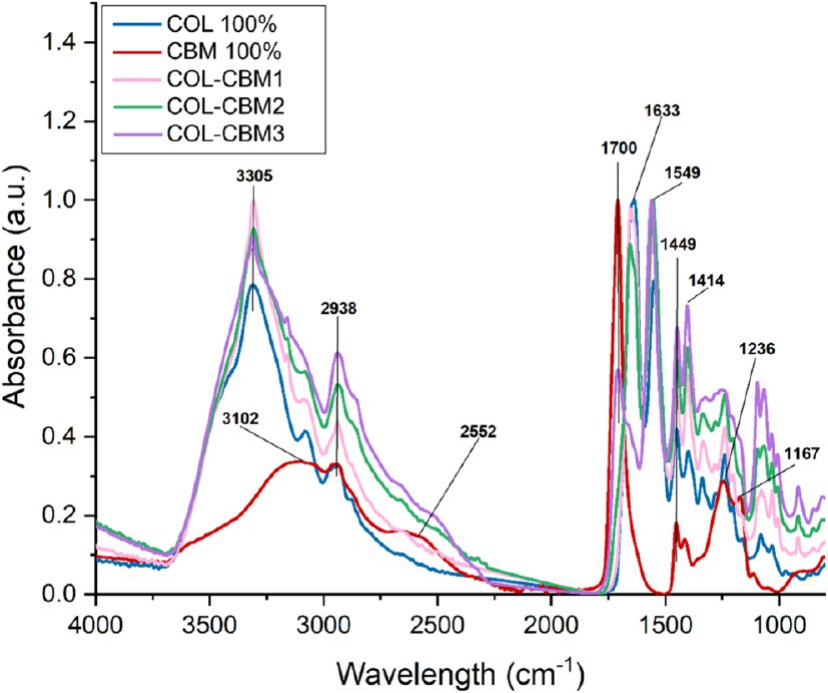
FT-IR spectra of COL 100%, CBM 100%, and COL-CBM samples.

The FT-IR spectrum of COL 100% shows collagen’s characteristic absorption bands, namely Amide A (3310 cm^–1^) related to N–H stretching vibration, Amide B (2920 cm^–1^) being assigned to the asymmetrical stretching of CH_2_, Amide I (1633 cm^–1^) related to the polypeptide backbone C=O stretching vibrations coupled with C–N stretching and N–H bending, Amide II (1549 cm^–1^), and Amide III (1236 cm^–1^). The presence of the Amide I absorption band is an indicator of hydrogen bond formation between N–H and carbonyl functional groups, and its presence confirms the integrity of the secondary structure of collagen. Amide II and Amide III absorption bands are linked to N–H bending vibrations coupled with C–N and C–H stretching.^49,58^ The FT-IR spectrum of HPMC also displays the specific absorption bands described in the literature, showing peaks at 3437, linked to −OH stretching vibrations, and at 2920 and 1062 cm^–1^ assigned to C–H and C–O stretching vibrations, respectively.^49,59^ The FT-IR spectra of COL-HPMC wafers show the representative peaks of both collagen and HPMC, with a decrease in intensity for collagen absorption bands correlated with the decrease in the percentage of collagen in the sample. The FT-IR spectrum of COL-HPMC1 showed high similarity to that of COL 100%, well correlated with the high collagen percentage in this sample (75%). Moreover, two peaks attributed to HPMC, situated at 2920 and 1062 cm^–1^, can be observed. COL-HPMC2 shows collagen characteristic absorption bands at 1648 cm^–1^ (Amide I – confirming the secondary structure) and 1549 cm^–1^ (Amide II), along with HPMC characteristic absorption bands at 3429, 2922, and 1062 cm^–1^. For the COL-HPMC3 sample, collagen-related bands are observed at 1633 and 1549 cm^–1^, along with HPMC-related bands at 3439, 2914, and 1064 cm^–1^.

The FT-IR spectrum of the CBM 100% sample also shows characteristic absorption bands, namely, a broad absorption band with peaks at 3102, 2938, and 2552 cm^–1^ assigned to −OH stretching vibrations and hydrogen bonding in the molecule, a high-intensity peak at 1700 cm^–1^ that could be attributed to C=O stretching from the carbonyl group, two peaks at 1449 and 1414 cm^–1^ related to −OH bending frequencies from the carboxylic groups, and C–O stretching vibrations. Peaks observed in this area could also be assigned to −CH_2_, −CH_3_, and −CH bending vibrations. Two other peaks, namely at 1236 and 1167 cm^–1^, could be attributed to the stretching vibration of the C–O–C from the ether groups.^60–62^

The FT-IR spectrum of the COL-CBM sample shows the characteristic absorption bands for collagen with similar intensities at similar wavenumbers. The COL-CBM1 spectrum displays absorption bands attributed to collagen at 3305 cm^–1^ (Amide A), 2938 cm^–1^ (Amide B), 1638 cm^–1^ (Amide I), 1549 cm^–1^ (Amide II), and 1236 cm^–1^ (Amide III). Also, two peaks are present at 1449 and 1414 cm^–1^, which could be an indicator for the −OH, −CH_2_, −CH_3_, and −CH bending vibrations assigned to the carbomer. The spectrum is very similar to that of 100% COL, indicating a high percentage of collagen present in the sample. The same trend is observed for COL-CBM2, with absorption bands attributed to collagen situated at 3305, 2936, 1640, 1549, 1230 cm^–1^, and carbomer characteristic bands at 1449 and 1414 cm^–1^. In the case of COL-CBM3, both peaks corresponding to collagen and carbomer can be observed, but with different intensities. The absorption bands situated at 3305 cm^–1^ (Amide A) and 2938 cm^–1^ (Amide B) are displayed in the spectra with a very high intensity, possibly coupled with the signal given by −OH stretching vibrations specific to the carbomer that arise at similar wavenumbers. Also, this could be attributed to intra- and intermolecular hydrogen bond formation. The peak present at 1700 cm^–1^ has a shoulder at 1655 cm^–1^, which could be due to the interaction between the two polymers and possible overlap with the carbomer’s characteristic absorption band attributed to C=O carbonyl stretching. Other highlighted peaks are present at 1550 cm^–1^ (Amide II), 1449, 1414, 1237, and 1093 cm^–1^. The latest specified absorption bands are all present with higher intensities than those observed in the FT-IR spectra of the rest of the samples.

Deconvolution of the Amide I spectral domain (around 1600–1700 cm^–1^) is often performed for analyzing the contribution of different secondary structure types for proteins. Being linked to the stretching vibration of carbonyl groups and the polypeptide backbone conformation, it is very sensitive and allows the identification and quantification of each secondary structure type based on different stretching vibrations of the carbonyl groups.^63–65^ Therefore, deconvolution of the spectral region corresponding to Amide I from the spectra of the collagen-based samples was performed. The band assignments to each type of secondary structure, their specific positions, and percentage areas are presented in Table 2. Band assignment has been performed based on data identified in the scientific literature.^66–71^

**Table 2:** Secondary Structure Content of Collagen-Based Samples after Deconvolution of the Amide I Region of the IR Spectra

sample	band assignment	ν (cm^–1^)	area (%)
COL 100%	β-sheet	1610	1
	random coil	1627	22
	α-helix	1654	74
	β-turn	1692	3
COL-HPMC1	β-sheet	1611	1
	random coil	1630	23
	α-helix	1658	73
	β-turn	1694	3
COL-HPMC2	β-sheet	1614	2
	random coil	1631	23
	α-helix	1659	73
	β-turn	1694	2
COL-HPMC3	β-sheet	1609	1
	random coil	1634	36
	α-helix	1660	48
	β-turn	1682	15
COL-CBM1	β-sheet	1614	1
	random coil	1629	21
	α-helix	1656	74
	β-turn	1694	4
COL-CBM2	β-sheet	1627	13
	random coil	1638	14
	α-helix	1659	33
	β-turn	1681	40

From the results presented in Table 2, it can be observed that for the COL 100% sample, the main contribution in the Amide I absorption band was assigned to the α-helix structural type (74%), followed by random coil (22%), β-turn (3%), and β-sheet (1%). These contributions are preserved in the combined samples with up to 50% HPMC, but change significantly for the COL-HPMC3 sample, recording a marked decrease in the α-helix structure type. In the case of the COL-CBM sample, it can be observed that the predominant α-helix structure and overall contribution of different secondary structure types are maintained in the COL-CBM1 sample but undergo a substantial change in the COL-CBM2 sample, indicating a more pronounced alteration of the secondary structure of collagen. The COL-CBM3 sample was excluded from this analysis due to the important changes that occurred in the position of the Amide I band for this sample, which can be observed in Figure 5a.

**Figure 5:**
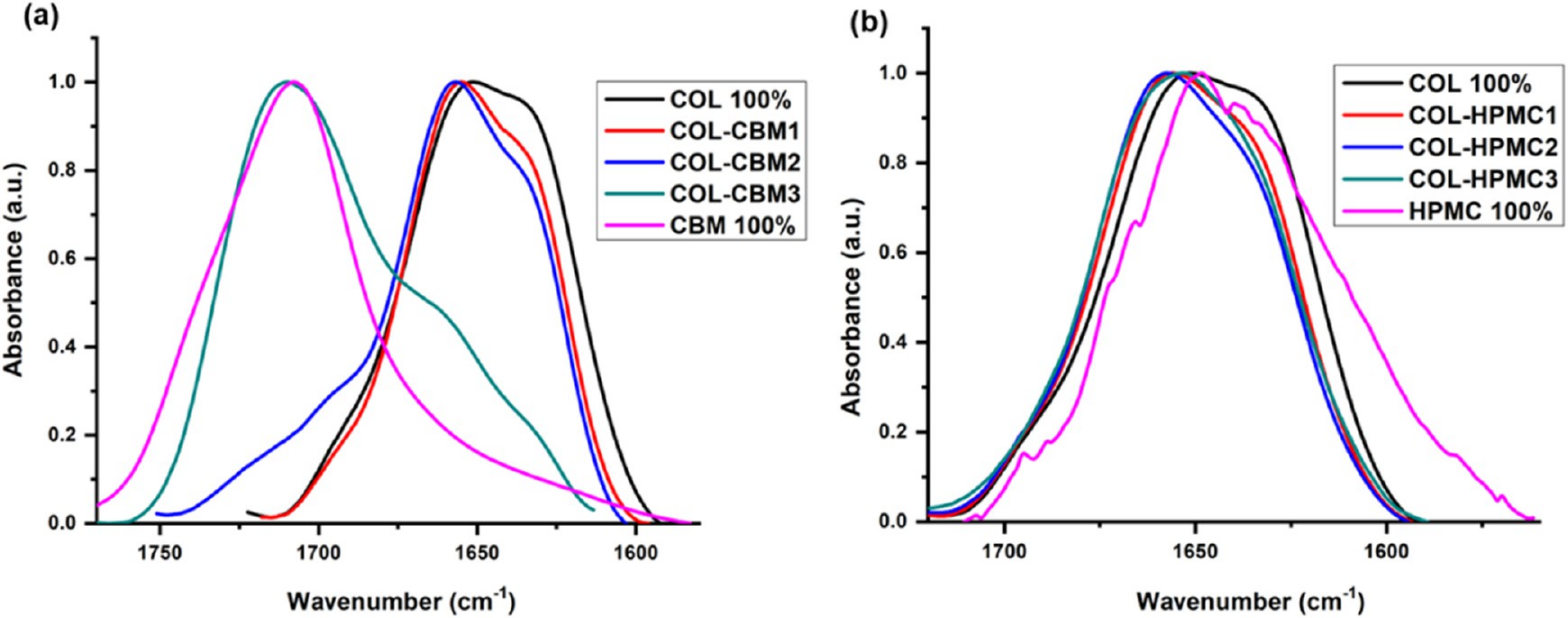
Overlay of FT-IR spectra monitored in the 1770–1580 cm^–1^ range (Amide I) for (a) COL-HPMC and (b) COL-CBM samples.

For the COL-HPMC series (Figure 5b), it can be observed that Amide I absorption bands have undergone a small shift in position toward higher wavenumbers, possibly related to changes that occur in the secondary structure of collagen. The same trend could be noted for the COL-CBM series. Nevertheless, the Amide I absorption band for the COL-CBM3 sample overlapped with the carbomer’s characteristic absorption band related to the stretching vibration of the C=O bond from the carboxyl group,^72^ and because of this, we were unable to accurately analyze the changes in the secondary structure of collagen by deconvoluting this absorption band.

### 3.3 Scanning Electron Microscopy (SEM)

SEM analysis was performed in order to investigate the morphology of the prepared freeze-dried wafers. The SEM images obtained for all nine samples are displayed in Figure 6. Based on the different polymer ratios present in each freeze-dried wafer, different microstructures can be noted. The COL 100% sample shows a microporous structure characteristic of collagen-based wafers,^73^ namely, large, multiple pores interconnected through entanglement of loose fibrils. In the HPMC 100% sample, a different, more compact, sheet-like structure with fewer pores can be observed, which can explain the low water uptake capacity of this sample. The CBM 100% wafer also displays a microporous structure, but with smaller pores and a much more compact polymeric network compared to the collagen-based wafer. Among the COL-HPMC samples, it can be noted that their morphology resembles that of the major constituent present in their composition based on the specified collagen-HPMC ratios. COL-HPMC1, containing 75% collagen, shows a microporous structure similar to COL 100%, but with smaller pores and a more compact fibril entanglement. In contrast, COL-HPMC3, in which the predominant component is HPMC, rather shows a sheet-like structure, also observed for HPMC 100%. COL-HPMC2 interweaves both types of structures, showing small interconnected pores separated by a compact fibril network. The same trend can be observed in the case of COL-CBM samples, which also resemble the structure of either collagen- or carbomer-based wafers. The COL-CBM1 sample is very similar to COL 100%, as expected, considering the high collagen ratio in its composition. As the percentage of CBM increases, the polymeric network becomes more compact, showing a larger number of pores but with smaller sizes. COL-CBM3 also resembles the morphology of the CBM 100% sample, with a slightly looser polymeric network.

**Figure 6:**
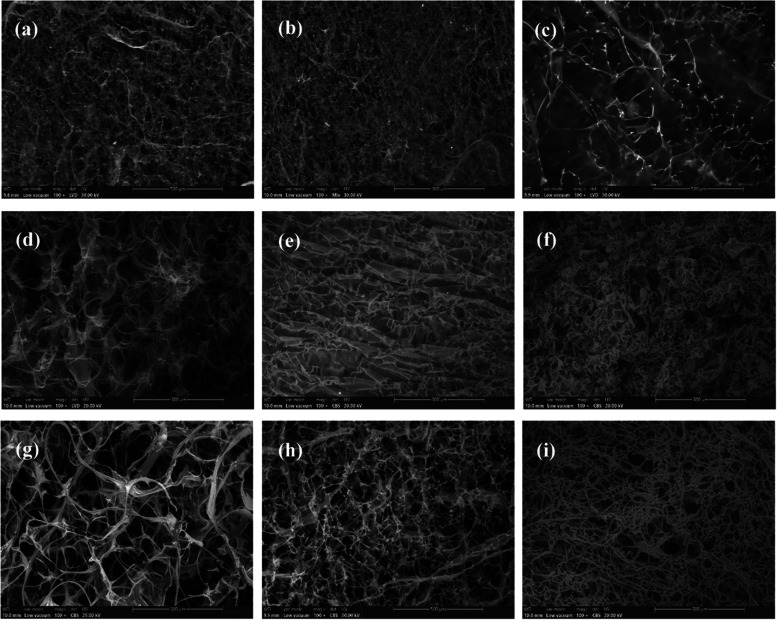
SEM images (100× magnification) of the prepared freeze-dried wafers: (a) COL-HPMC1, (b) COL-HPMC2, (c) COL-HPMC3, (d) COL 100%, (e) HPMC 100%, (f) CBM 100%, (g) COL-CBM1, (h) COL-CBM2, and (i) COL-CBM3.

### 3.4 Thermogravimetric Analysis (TGA)

TGA was performed in order to investigate the thermal stability of the obtained wafers by determining the weight changes that occurred with the increase in temperature. These mass changes are directly related to the degradation profile of the sample.^74,75^ In the present study, TGA was used in order to assess the thermal stability of the obtained freeze-dried wafers and to investigate the potential effects generated by adding HPMC or CBM on the thermal stability of collagen. Figure 7 displays the TGA curves illustrating the freeze-dried wafer heat degradation profiles in nitrogen conditions.

**Figure 7:**
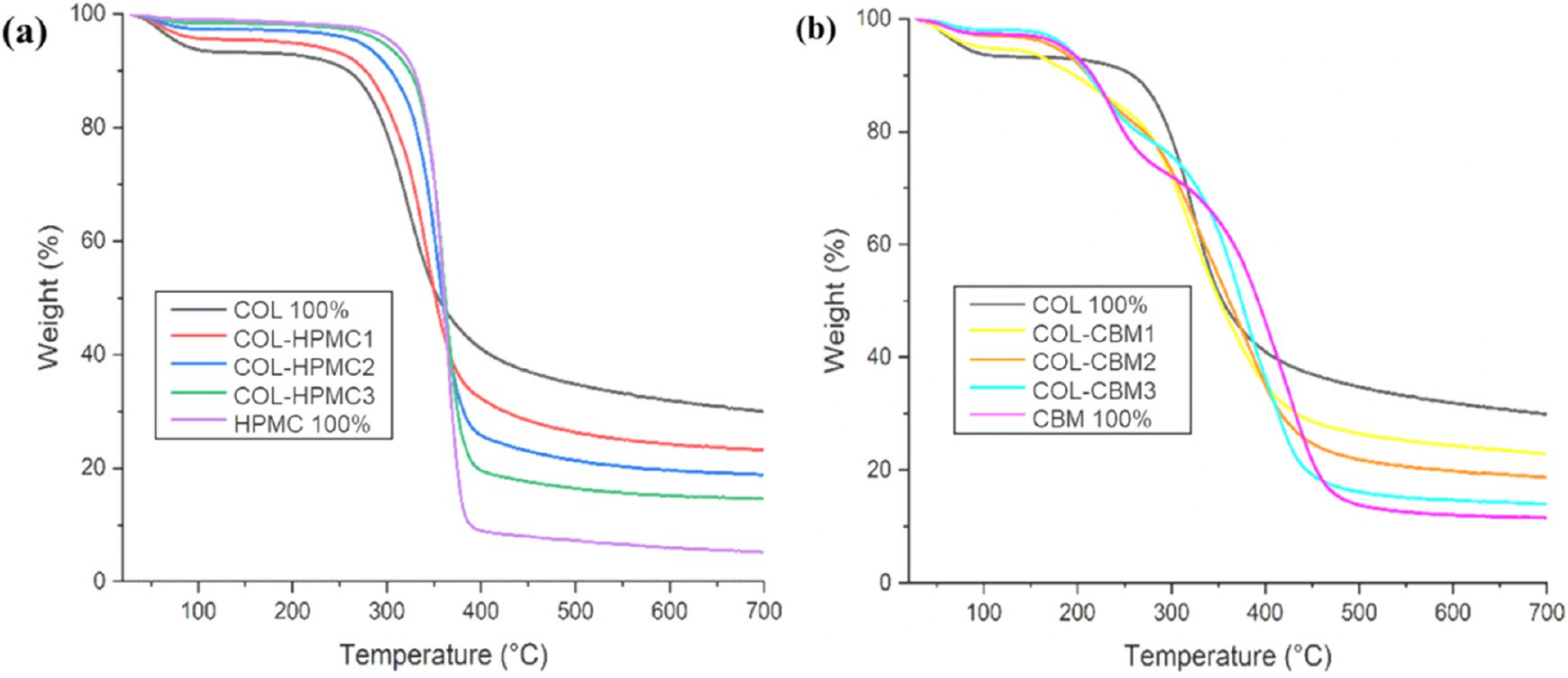
TGA thermograms of (a) COL-HPMC and (b) COL-CBM samples.

The thermograms displayed in Figure 7a show that the addition of HPMC improved the thermal stability of the samples up to 350 °C. TGA thermograms of the COL-HPMC samples revealed a multiple-step degradation for each sample, with two main thermal events being highlighted. These results are in agreement with previous literature data.^49^ The first thermal transitional change occurs between 28 and 200 °C and might be caused by the loss of water from the sample. Hydrogen bonds between and within molecules, as well as water that is hydrogen-bound, all contribute to the stability of collagen’s triple-helical structure.^76–78^ Thus, the acquired results show some modifications of the collagen macromolecule’s secondary structure at this stage, with a registered weight loss of 7% for the COL 100% sample. Subsequently, given the correlation between the increase in the proportion of HPMC and the decrease in weight loss for the combined samples (COL-HPMC), it could be assumed that the presence of HPMC in collagen-based samples improved their thermal stability at this stage. The second thermal event, which is related to an irreversible denaturation process and the breakdown of collagen’s triple-helical structure, transitioning to a random coiled structure, was observed in the temperature range of 200–700 °C.^53,78^ COL 100% and COL-HPMC1 (75% collagen and 25% HPMC) showed the highest thermal stability in this temperature range. This thermal event could also be associated with the oxidative degradation of cellulose ethers, resulting from two concurrent processes, namely, dehydration and demethylation.^49,79^ This could explain the increased weight loss registered for the samples with increased HPMC content. A small weight loss (2–5%) can be observed in the terminal region of this second temperature range (500–700 °C), which could be associated with the evaporation of residual components from all the samples. The TGA results for the COL-HPMC samples are presented in Table 3.

**Table 3:** Thermogravimetric Parameters of the COL-HPMC Samples

	step 1		step 2		
samples	*T*, °C	weight loss, %	*T*, °C	weight loss, %	residual mass, %
COL 100%	28–200	7	200–700	63	30
COL-HPMC1		5		72	23
COL-HPMC2		3		78	19
COL-HPMC3		2		84	14
HPMC 100%		2		93	5

As can be seen from the results obtained in the first temperature range (28–200 °C), the weight loss percentage decreased with the increase in HPMC content in the sample, indicating that COL-HPMC samples were more stable against heat degradation than COL 100% up to 200 °C. Regarding the COL-CBM samples, it can be observed in the thermograms of Figure 7b that the addition of CBM did not improve the thermal stability of the samples. TGA thermograms of the COL-CBM samples displayed a multiple-step denaturation profile for each sample, in this case, with three main thermal events being highlighted, similar to other findings identified in the literature.^80^ Thermogravimetric parameters of the analyzed COL-CBM samples are presented in Table 4.

**Table 4:** Thermogravimetric Parameters of the COL-CBM Samples

	step 1		step 2		step 3		
samples	*T*, °C	weight loss, %	*T*, °C	weight loss, %	*T*, °C	weight loss, %	residual mass, %
COL 100%	28–200	7	200–350	42	350–700	21	30
COL-CBM1		10		40		27	23
COL-CBM2		8		38		35	19
COL-CBM3		7		31		48	14
CBM 100%		7		29		52	12

Thus, considering the results obtained for all the analyzed samples, it may be concluded that the addition of CBM in the collagen matrix alters the native collagen conformation in a more significant way, while the addition of HPMC led to improved thermal stability of the collagen-based wafers in the temperature range of interest for this study. These conclusions are in agreement with the circular dichroism results.

### 3.5 Contact Angle Measurements

Contact angle measurements were performed in order to assess the wettability behavior of the freeze-dried wafers. Distilled water was used in order to evaluate the surface hydrophobicity, with the solid surface being considered hydrophobic if the water contact angle exceeds 90° and hydrophilic if the contact angle is smaller than 90°.^81^ Hydrophilicity is an important characteristic of wafers intended for mucosal drug delivery, considering the fact that they should quickly hydrate and swell upon administration in order to adhere to the mucosa and release the incorporated drug.

Given the fact that wafers, also known as sponges, are porous matrices that possess absorbing characteristics and a heterogeneous surface, a static contact angle cannot be obtained. In this case, the contact angle will decrease as imbibition occurs, with the drop being absorbed by the spongious matrices. Therefore, the contact angle for porous materials is dynamic and multiple, and decreasing values will be obtained in a specified time frame.^82,83^ In order to evaluate the wettability of the prepared freeze-dried wafers, we used the mean values. Three measurements were performed for each sample, and the results are presented in Figure 8.

**Figure 8:**
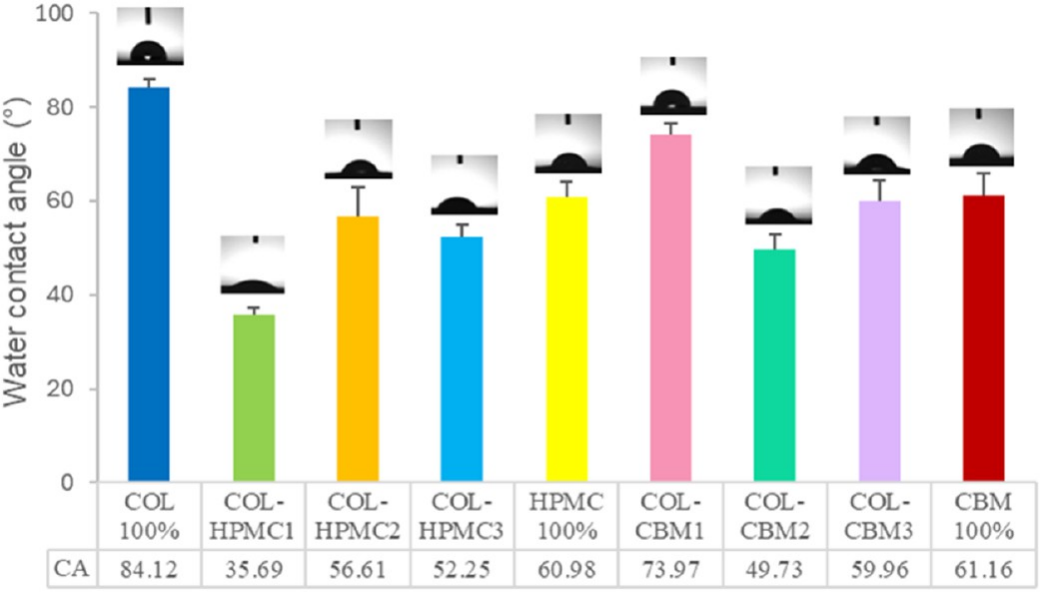
Comparative plot of mean contact angle values obtained for freeze-dried wafers.

As can be seen from the results, the highest contact angle was obtained for the COL 100% sample, namely 84.12°. All samples displayed contact angle values <90°, indicating hydrophilic behavior for the freeze-dried wafers, which makes them good candidates for mucosal drug delivery. COL-HPMC1, with a percentage composition of 75% collagen and 25% HPMC, showed the lowest contact angle value among all samples, displaying the highest hydrophilicity. HPMC 100% showed a mean contact angle value of 60.98°, which correlates well with the hydrophilic character of this polymer. COL-HPMC2 and COL-HPMC3 showed similar contact angle values of 56.61° and 52.25°, respectively.

Regarding the COL-CBM samples, our results confirmed the hydrophilic character of these samples, with all average contact angle values being <90°. The highest contact angle in this group was obtained for COL-CBM1 (73.97°), probably because of the high collagen content in this sample. Similar mean contact angle values were obtained for the COL-CBM3 and CBM 100% samples (59.95 and 61.16°, respectively). These samples had the highest Carbomer 940 contents of 75 and 100%, respectively. The COL-CBM2 sample, containing 50% collagen and 50% Carbomer 940, displayed the lowest value of 49.72°, possibly determined by the interaction between the two polymers. The obtained results show good hydrophilicity for the analyzed samples, even if a specific trend could not be highlighted, probably due to the heterogeneous surface of the samples and different pore sizes and distributions.

### 3.6 Water Uptake Capacity of Freeze-Dried Wafers

In order to evaluate the swelling behavior of the obtained freeze-dried wafers, the water uptake capacity was investigated. The water uptake capacity (g/g) of the COL-HPMC wafers, investigated throughout the 72 h test interval, is displayed in Figure 9. Among all tested wafers, COL 100% exhibited quick water absorption, followed by reaching an equilibrium state, with the highest water uptake capacity of 34.10 g/g being reached 2 h postimmersion in water. The wafer sample maintained its structural integrity in a hydrated form during the whole 72 h test interval. A similar behavior was observed for COL-HPMC1, with the highest water uptake capacity among the samples containing collagen and HPMC (40.00 g/g). Along with COL 100%, COL-HPMC1 retained its structure in hydrated form until the end of the experiment, probably due to the high collagen content (75%), making it a great candidate for prolonged drug release.

**Figure 9:**
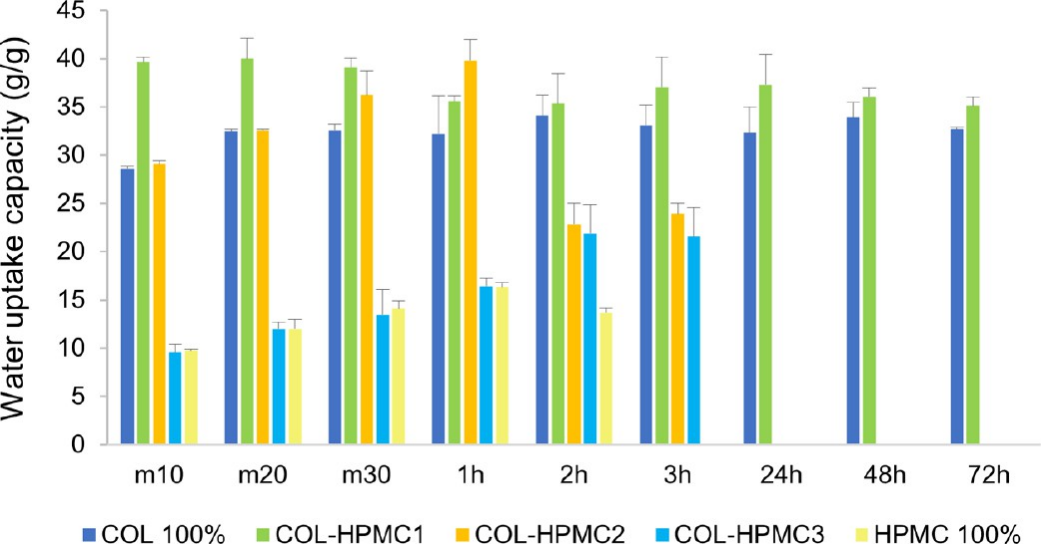
Water uptake capacity (g/g) of collagen/HPMC-based wafers throughout the 72 h test interval.

The HPMC 100% sample showed a different behavior, namely, absorbing a lower amount of water, with the highest water uptake capacity of 16.33 g/g, recorded at 1 h postimmersion. Shortly after the first 2 h, complete rehydration of the wafer took place, and the sample transformed into the corresponding hydrogel. These results are consistent with HPMC’s expected behavior, being a water-soluble cellulose derivative with marked hydrophilic character and good swelling behavior.^84,85^ COL-HPMC2 and COL-HPMC3 showed an intermediate behavior between COL 100% and HPMC 100%, with water uptake capacity decreasing with the increase in the HPMC ratio in the two samples. Both wafers were rehydrated within 24 h of postimmersion in distilled water, generating the corresponding hydrogels.

Regarding the water uptake capacity of COL-CBM wafers, a different behavior can be noted. The water uptake capacity of the COL-CBM samples is presented in Figure 10.

**Figure 10:**
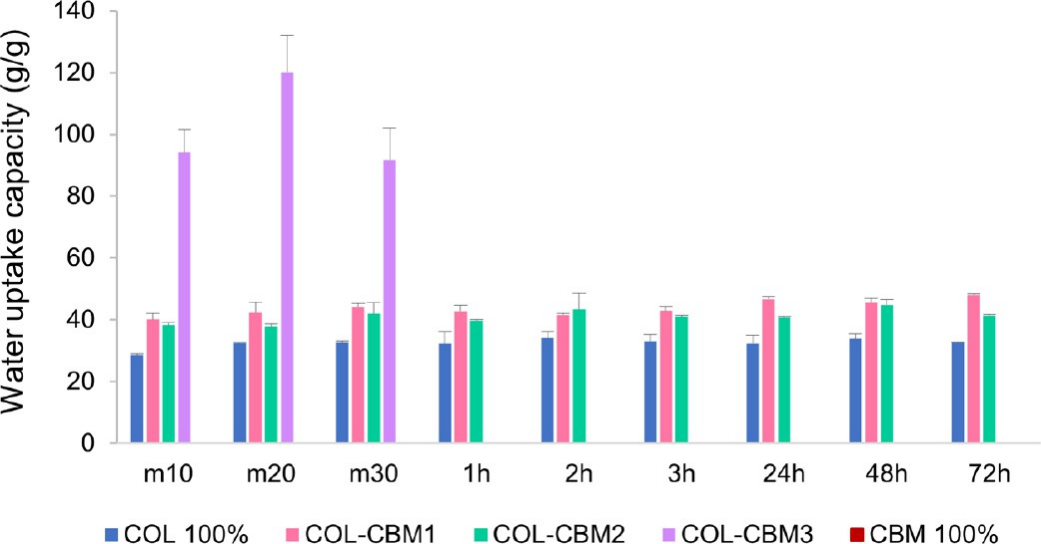
Water uptake capacity (g/g) of collagen/CBM-based wafers throughout the 72 h test interval.

The CBM sample hydrated almost immediately upon immersion in distilled water. The COL-CBM3 sample showed a marked water uptake capacity, reaching a value of 94.08 g/g at 10 min after hydration, followed by an even higher value of 120.036 g/g reached at 20 min postimmersion, but quickly losing its structural integrity within the first 30 min of the experiment. The rapid loss of structural integrity could be due to the high CBM percentage in this sample, namely, 75%. This wafer shows similar behavior to CBM 100%. COL-CBM1 and COL-CBM2 freeze-dried wafers showed the highest water uptake capacity of 48.125 and 44.90 g/g, respectively, and maintained their structure throughout the whole testing interval. This could be due to the high collagen percentage in this sample, namely, 75% for COL-CBM1 and 50% for COL-CBM2.

The water uptake capacity highlighted the different swelling behaviors for the COL-HPMC and COL-CBP samples. This analysis confirmed the hydrophilic characteristics of the wafers. Both HPMC and Carbomer 940 showed a tendency to hydrate quickly and turn into the corresponding hydrogel, with the difference being the amount of water absorbed, which was smaller in the case of HPMC and significantly higher for Carbomer 940.

### 3.7 Biocompatibility Assessment of Freeze-Dried Wafers

It is known that collagen, as a natural extracellular matrix protein, provides an exceptional biological setting that supports cell adhesion, proliferation, and migration.^30^ HPMC is known for its biocompatibility and ability to form hydrogels with favorable water retention properties, which could support cell proliferation.^86^ On the other hand, CBM is a polymer of synthetic origin, broadly used for its potency as a gelling agent, especially in the development of pharmaceutical and cosmetic products, and in the food industry,^22^ having also good biocompatibility.^22^ Thus, it is to be expected that the blend of collagen with mucoadhesive polymers such as HPMC or CBM could support cell viability and proliferation due to their physicochemical and biological properties. Therefore, the biocompatibility of freeze-dried wafers was assessed by incubating human adult fibroblasts with the extracts obtained as described above. The impact of these mixtures on cell viability, morphology, and migration capacity was evaluated, along with their ability to induce a chemotactic effect.

As shown in Figure 11, except for CBM, which had a strong cytotoxic effect, all of the other tested samples supported the viability of fibroblasts. Moreover, the results showed that, after 72 h of incubation, HPMC and COL-HPMC samples induced increased cell proliferation, with COL-HPMC3 being the most effective in this regard. Thus, all these biomaterials could release factors that might promote fibroblast proliferation.

**Figure 11:**
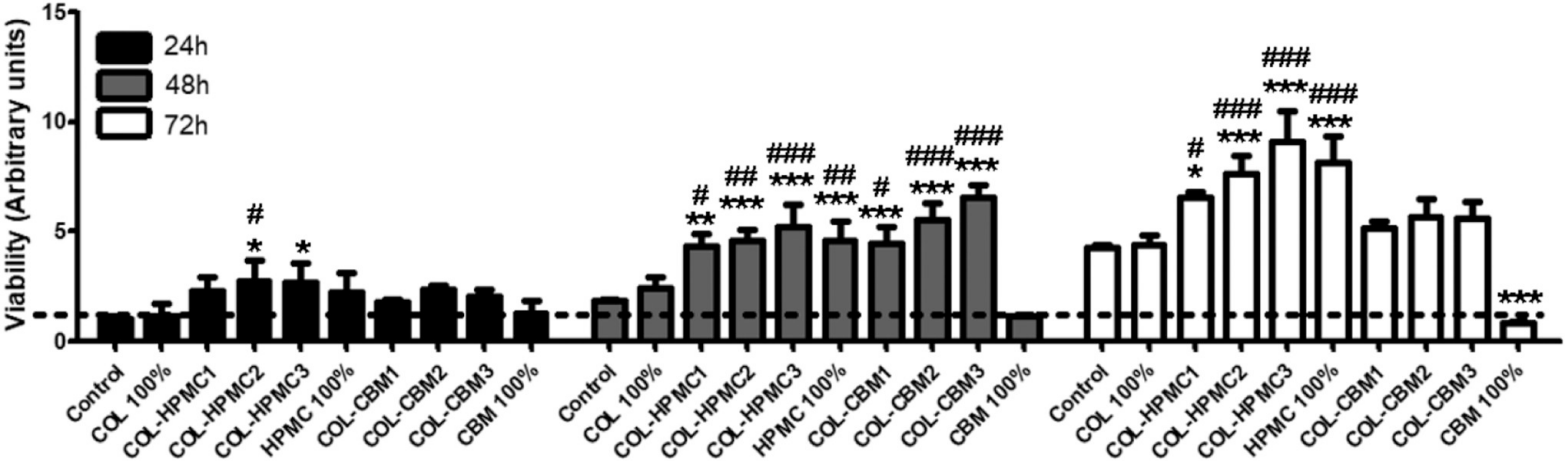
XTT assay showing the viability of human adult fibroblasts after 24, 48, and 72 h of incubation in the presence of freeze-dried wafer extracts (*p < 0.05, **p < 0.01, ***p < 0.001 versus control at each time point; ^#^ *p* < 0.05, ^##^ *p* < 0.01, ^###^ *p* < 0.001 versus COL at each time point).

Next, we evaluated the impact of the freeze-dried wafers on the fibroblasts’ capacity to migrate, as the migration of cells at the lesion site is an important step in the wound healing process. Figure 12 shows that, as in the case of viability data, except for CBM 100%, none of the tested biomaterials had any inhibitory effect on cell motility. Statistical significance was obtained for COL-HPMC1, COL-HPMC3, HPMC, and COL-CBM1, which had an average coverage of 48, 59, 47, and 55%, respectively, of the scratched area versus the positive control (cells incubated in complete medium). However, only COL-HPMC3 induced migration that was statistically significant versus COL: 59% versus 41%. These data suggested that, in this case, HPMC addition improves the properties of the COL 100 sample. As a negative control, cell culture media without serum was used.

**Figure 12:**
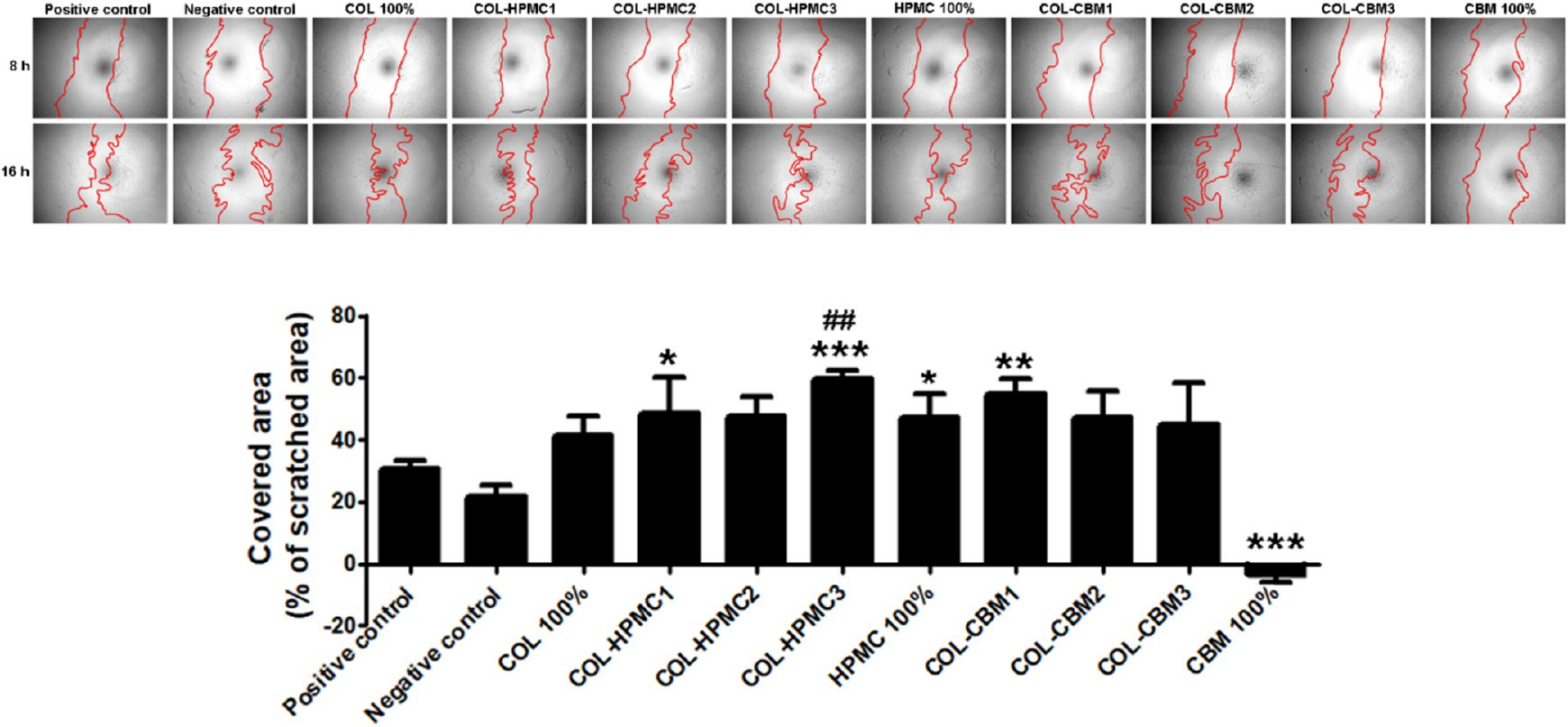
In vitro scratch test showing the impact of collagen-based freeze-dried wafers on fibroblast motility. Upper panel: phase-contrast microscopy showing the scratched area at 0 and 16 h later (magnification 5×); lower panel: the quantification of covered area as percentage of the initial scratched area where positive and negative controls are serum and serum-free medium (*p < 0.05, **p < 0.01, ***p < 0.001 versus the positive control; ^##^ *p* < 0.01 versus COL).

The data obtained for the evaluation of cellular migration capacity in the presence of the tested biomaterials were validated by cell morphology evaluation because the cytoskeleton has a major impact on the mechanical properties of cells and is responsible for intercellular mechanical and functional interactions. Images of fluorescence microscopy, presented in Figure 13, showed that after 24 h of incubation with the freeze-dried wafer extracts, the cells spread uniformly on the culture plates in all tested samples, and after 72 h they reached approximately 80% confluency, except for the CBM 100% sample, which was shown by the previous methods to be cytotoxic. Fibroblasts cultured in the presence of all samples had a spindle-shaped, elongated phenotype with protrusions, suggesting that the migratory capacity remained unaffected after 72 h of incubation in the presence of biomaterial extracts.

**Figure 13:**
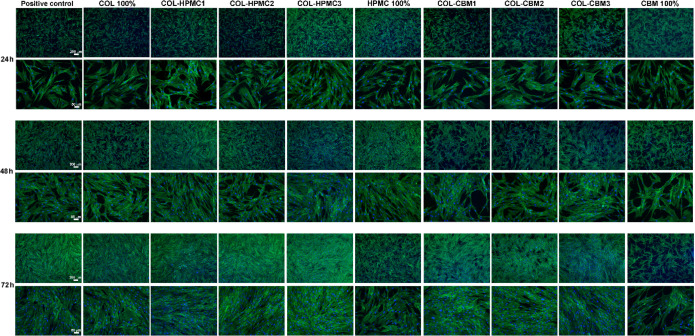
Images of fluorescence-based staining of the actin cytoskeleton (green fluorescence) and nucleus (blue). For evaluation of cell density, images are shown in the upper panels of each time point, with lower magnification (bar = 200 μm), while the cytoskeleton organization and F-actin stress fibers are shown in the lower panels with higher magnification (bar = 50 μm).

Given the fact that COL-HPMC3 samples had an improved effect on cell proliferation and migration compared to all other samples and was significantly better than COL alone, we next questioned if it could also have a chemotactic effect toward fibroblasts, which would be an additional advantage in the wound healing process. As seen in Figure 14, at 3 h, COL and COL-HPMC3 induced cell migration through the porous membrane at the same level as the complete medium. However, at a longer time (6 h), there appears to be a difference between the capacity of various samples to chemoattract fibroblasts. Thus, COL-HPMC3 had the highest cell index at 6 h, compared to both COL and serum control, although not statistically significant. This suggests that COL-HPMC3 freeze-dried wafers could have a certain chemoattractive effect toward fibroblasts.

**Figure 14:**
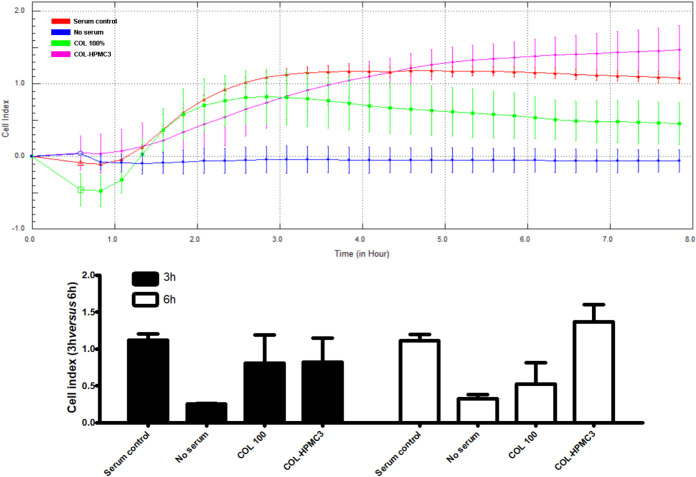
Time-dependent assessment of chemotactic migration of human adult fibroblasts. The lower panel illustrates the migration index at 3 h versus 6 h.

## 4 Conclusions

Hydrogels based on different concentrations of collagen and two mucoadhesive polymers were lyophilized, and spongious forms referred to as freeze-dried wafers were obtained. The results of circular dichroism of hydrogels showed that the addition of Carbomer 940 led to more significant alterations in collagen’s triple-helical structure than those induced by the addition of HPMC. The obtained FT-IR spectra displayed characteristic peaks for all polymers. TGA analysis showed that HPMC improved the thermal stability of the samples in the temperature range of interest, while the addition of Carbomer 940 led to decreased thermal stability. SEM images revealed the combined morphological characteristics of both collagen and mucoadhesive polymers for the composite wafers. Water uptake capacity evidenced different swelling behaviors for the COL-HPMC and COL-CBM samples. The analysis confirmed the hydrophilic characteristics of the wafers, also highlighted by water contact angle measurements, with values <90° obtained for all tested samples. Except for CBM 100%, all samples showed good biocompatibility, supporting cell viability and motility. The addition of HPMC to the samples induced an increase in cell proliferation, with the best results for COL-HPMC3, which further demonstrated a certain chemoattractive effect toward human fibroblasts. Considering all the obtained results, COL-HPMC samples had a better performance throughout the analyses, compared to COL-CBM samples, and could represent a starting point for future studies. However, a proper balance must be established between the physicochemical, mechanical, and biological properties of the prepared biomaterials in order to develop some effective and biocompatible drug delivery systems intended for mucosal administration.
